# A systematic search for discriminating sites in the 16S ribosomal RNA gene

**DOI:** 10.1186/2042-5783-4-2

**Published:** 2014-01-27

**Authors:** Hilde Vinje, Trygve Almøy, Kristian Hovde Liland, Lars Snipen

**Affiliations:** 1 Department of Chemistry, Biotechnology and Food Sciences, Norwegian University of Life Sciences, Ås N-1432, Norway; 2 Nofima AS, Osloveien 1, Ås 1430, Norway

## Abstract

**Background:**

The 16S rRNA is by far the most common genomic marker used for prokaryotic classification, and has been used extensively in metagenomic studies over recent years. Along the 16S gene there are regions with more or less variation across the kingdom of bacteria. Nine variable regions have been identified, flanked by more conserved parts of the sequence. It has been stated that the discriminatory power of the 16S marker lies in these variable regions. In the present study we wanted to examine this more closely, and used a supervised learning method to search systematically for sites that contribute to correct classification at either the phylum or genus level.

**Results:**

When classifying phyla the site selection algorithm located 50 discriminative sites. These were scattered over most of the alignments and only around half of them were located in the variable regions. The selected sites did, however, have an entropy significantly larger than expected, meaning they are sites of large variation. We found that the discriminative sites typically have a large entropy compared to their closest neighbours along the alignments. When classifying genera the site selection algorithm needed around 80% of the sites in the 16S gene before the classification error reached a minimum. This means that all variation, in both variable and conserved regions, is needed in order to separate genera.

**Conclusions:**

Our findings does not support the statement that the discriminative power of the 16S gene is located only in the variable regions. Variable regions are important, but just as many discriminative sites are found in the more conserved parts. The discriminative power is typically found in sites of large variation located inside shorter regions of higher conservation.

## Background

The use of stable parts of the genomic content as an evolutionary marker was a breakthrough for microbial studies in the 1980s [1,2]. The 16S small ribosomal subunit gene (16S rRNA) is today considered the gold standard for phylogenetic studies of microbial communities and for assigning taxonomic names to bacteria [3-5]. There are several properties of the 16S gene that has made it useful as a taxonomic target. First, the 16S gene is present in all bacteria. Second, it contains regions resistant to prokaryotic evolution [2]. This has made it possible to recognize the 16S without too much problems in most genomes. Third, and most important to this study, the 16S gene also includes some variable regions in between the more conserved parts. Nine such regions were once identified and named V1-V9 [6] from the sequence data available at that time. Based on the data sets of those days, it was concluded that the conserved regions are too conserved to be useful for discriminating between taxa, and that the variable regions are the key to classification of prokaryotes. Some later studies [7,8] have also confirmed these results, establishing a dogma in the use of 16S sequence data: The information separating taxa is found in the variable regions of the 16S gene.

The location of the variable regions, and implicitly the conserved parts flanking them, has been based on some multiple alignment of more or less full-length 16S genes. Van de Peer et al. [6] used distances between sequences together with the specific nucleotide substitution rate for each position to identify the variable regions. Another approach is to compute the entropy for each position in the alignment [9], and conserved/variable regions correspond to low/high entropy.

The conserved parts are used to locate the marker gene, either *in silico* in a sequence of genomic DNA, or more commonly, *in situ* by polymerase chain reaction (PCR) amplification [10] based on primers matching these conserved parts. The first sets of primers were named according to their positions on *Escherichia coli* 16S rRNA [11]. Over the years many publications have been devoted to improving these primers [12,13].

In recent years it has been discovered that the conserved parts are not in fact as conserved as once conceived, and that there are really no such thing as universal PCR-primers that will sample equally well in all branches of the tree-of-life [14-16]. A recent study by Mizrahi-Man *et al.*[17] consider, among other things, how well the various variable regions are suited for classification. Still, these investigations all have in common that they first fix a set of primers, and then look at the regions between the primer-matching sites to see if the corresponding sub-sequences discriminate well or not. In this article we want to examine the whole length of the 16S gene, and to see if mining in the huge set of available 16S sequences can tell us something about where the discriminating sites are located, without any constraints with respect to primer matching sites.

We approach this problem by classifying the 16S sequences using a multivariate method and data consisting of multiple alignments. We conduct a systematic search for the best discriminative sites along the alignments. We use high-quality data from the databases Greengenes [18], the Ribosomal Database Project (RDP) [19] and SILVA [20]. The aim of this study is to investigate where the most discriminative sites in the 16S marker gene are located, more specifically if they correspond to variable or conserved regions.

## Methods

### Data

Data were downloaded from three databases; Greengenes [21], RDP [22] and SILVA [23]. From Greengenes we downloaded the alignment of isolated named strains, containing 117 101 sequences over 7682 positions. From RDP we downloaded all bacterial sequences marked as good quality and with at least 1200 bases which resulted in an alignment containing 1 151 913 sequences over 22 721 positions. From SILVA we downloaded the archived alignment named SSURef_111_NR_tax_silva_trunc_aligned containing 286 858 sequences over 45 984 positions.

From all alignments we discarded sequences less than 1200 bases long, sequences having alien characters (not A,C,G,T or -) and sequences not classified to one of the 2074 bacterial genera listed in the List of Prokaryotic names with Standing in Nomenclature (LPSN, http://www.bacterio.cict.fr/). We also discarded duplicated sequences. This resulted in a reduced alignment of high-quality data from each database, see Table 1.

**Table 1:** Overview of data

**Database**	**Downloaded**	**Filtered**	**Intersection**
Greengenes	117101×7682	74928×3664	12362×3166
RDP	1151913×22721	135120×16686	12362×4084
SILVA	286858×45984	111914×13172	12362×4230

Each cell shows the number of sequences × the number of positions of each alignment. Downloaded are the original alignments, Filtered means after filtering of high-quality data (see text) and Intersection is the subset of sequences common to all databases.

**Figure 1:**
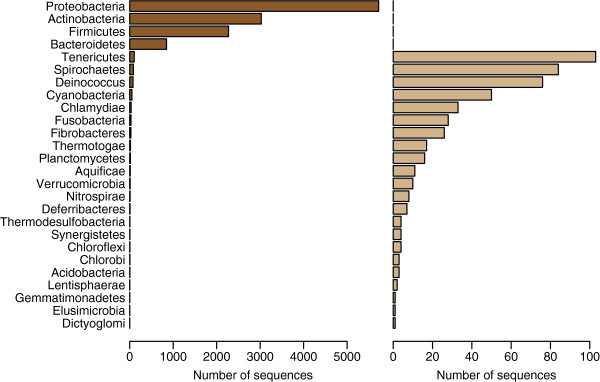
**Distribution of phyla in data set.** The final 12362 sequences are classified into 26 different phyla in the data set. The left panel shows the distribution among these phyla. The right panel gives a more detailed picture for the smaller numbers, with the four most common phyla ignored.

Finally, we focused on the subset of sequences found in all three databases, i.e. the intersection between the databases. In order to obtain a consensus-based class label for all sequences, we also discarded sequences assigned to different genera in the three databases. We were then left with 12362 sequences found in all three databases, see Table 1. For each of these sequences both the assigned phylum and genus were recorded as two alternative class labels. Figure 1 shows the distribution of phyla in this data set.

When performing the systematic search for discriminating sites, phyla with less than 25 sequences were discarded, leaving us with data for 11 phyla and a total of 12270 sequences in the data set. When using genus as response, we required at least 10 sequences in each genus, resulting in 198 distinct genera (9948 sequences).

### Entropy

To relate sites in the three alignments to each other, and to conserved/variable regions, we computed the entropy for each site in each alignment. This approach has also been used in previous studies (e.g. [9]). For all three alignments all sites consisting of less than 30 A, C, G and T were discarded as these provided too little data. At each remaining site *k* we computed the entropy

(1) $$H_{k}=-\overset{4}{\underset{i=1}{\sum }}\;p_{i}\log(p_{i})$$

where *p*_1_,*p*_2_,*p*_3_ and *p*_4_ are the empirical proportions of the four bases appearing at position *k*.

**Figure 2:**
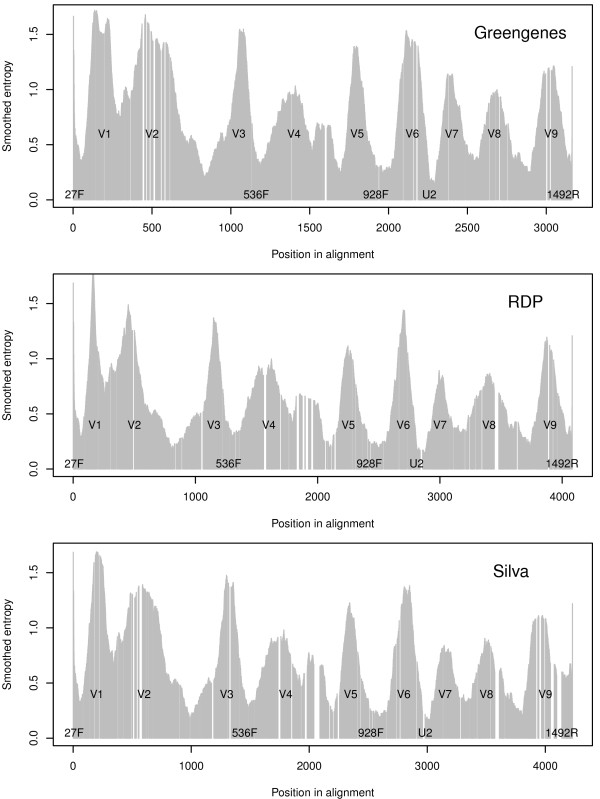
**Smoothed entropy.** The three panels show the smoothed entropy for the Greengenes, RDP and SILVA alignments covering the same 12362 sequences in this study. Positions with less than 30 bases have no entropy here, hence the ’holes’ at some positions. Notice the difference in the number of positions, Greengenes being the shortest and SILVA the longest alignment. The nine variable regions V1,..., V9 are indicated for each alignment. Five examples of primers (27F, 536F, 928F, U2 and 1492R) used for PCR amplification of 16S are also marked along the position axis, indicating where they frequently match.

In order to visually identify the regions of high/low entropy, this entropy was smoothed across positions using a centered moving average of length 51. Figure 2 is a visualisation of this from the three different alignments. Note, the position specific entropy from (1) was used in the subsequent analysis, the smoothing was only used to illustrate.

### Site selection algorithm

In order to search for discriminating sites along the 16S alignments in a systematic way, we implemented a supervised learning approach. The input data to the supervised learning method are one of the three alignments previously described and the class-labels for each sequence in the alignment. We have used the Partial Least Squares (PLS) method [24], which is one in a long list of supervised learning methods. PLS is well established and has been used in many bioinformatics applications, also for the analysis of sequence data [25,26]. PLS is especially applicable when there are many correlated explanatory variables. This will typically be the case for the present data since the explanatory variables are in our case the sites in the alignments, and many sites along the alignment will have similar base compositions giving high correlations.

All three alignments were considered one at a time. Each site in the alignment contains a column with the symbols A,C,G,T or -. In order to use the supervised learning method we coded each symbol into a row-vector of five binary values. The symbol A was coded as (1,0,0,0,0), C as (0,1,0,0,0), G as (0,0,1,0,0), T as (0,0,0,1,0) and the indel - as (0,0,0,0,1). Thus, each *N*×1 column of symbols in the alignment gives rise to a *N*×5 matrix of binary values to be used in the PLS-algorithm. Where *N* is the number of sequences. We use the term *variable* instead of *site* below, but each site actually gives rise to five numerical (binary) variables.

The response variable is in this case the class labels, and this was also coded in a similar way, using one bit for each class. As an example, when using phylum as response, the single *N*×1 column containing 11 different phyla was translated into an *N*×11 matrix of binary values, where Proteobacteria corresponds to (1,0,0,0,0,0,0,0,0,0,0), Firmicutes to (0,1,0,0,0,0,0,0,0,0,0) etc.

Being a multivariate method, PLS finds combinations of the explanatory variables giving the minimum classification error. These combinations are referred to as PLS components. In principle, all explanatory variables are included, and given more or less weight in the components. Variable selection means we intend to select only a subset of the original explanatory variables, and then combine these to achieve the best possible discrimination. There are many approaches to variable selection under the PLS paradigm [27], and for this application we have chosen the Selectivity Ratio (SR) score as the criterion. The SR-score is the ratio of explained variance to residual variance for each variable. This represents a measure of the ability to discriminate between the classes. High SR-score for a variable means it contains information about the classes and can discriminate between these in a good way [28].

The site-selection algorithm contained the following steps: 

•A 10-fold cross validation was first used to find the optimal number of PLS-components needed to classify the given response with the minimum obtainable error.

•A PLS regression model was fitted to the full data set, with the fixed number of components from Step 1, to obtain regression coefficients for all explanatory variables. For every explanatory variable the selectivity ratio.

•For every explanatory variable the selectivity ratio was calculated based on the regression coefficients from 2. Due to the coding, each site in the alignment corresponds to five SR-scores. The maximum of these five SR-scores was used as a site specific SR-score.

•These site specific SR-scores were sorted in descending order; the largest SR-score corresponding to the most interesting sites. One by one the sites were included in the final model, and a 10-fold cross validation was again conducted to estimate a classification error. The final choice of how many sites to include was based on this classification error.

## Results and discussion

We extracted 12362 unique sequences from the three databases Greengenes, RDP and SILVA, all having at least 1200 bases, no alien characters, found in all three databases and with identical assignment to genus. This consensus data set must be considered a high-quality data set for 16S sequences, and an overview is given in Table 1. The three databases provide alignments of these sequences, and Figure 2 shows the smoothed entropy in each case. The three alignments differ, specifically the number of sites are different, which is due to a differing number of gaps introduced. However, the smoothed entropy shows a fairly similar pattern in all cases, and nine peaks can, with some good will, be identified. We emphasize that the grey bars in Figure 2 shows the *smoothed* entropy in order to display the regions. The actual entropy at the various sites fluctuates much more, as we will come back to below.

Instead of focusing on conserved or variable sites, we used the PLS supervised learning method to extract the sites giving the best possible discrimination regardless of where they may be along the alignment. First, we used phylum as response, i.e. there are 11 distinct classes, and for each of the three alignments (Greengenes, RDP and SILVA) we employed the site selection algorithm.

**Figure 3:**
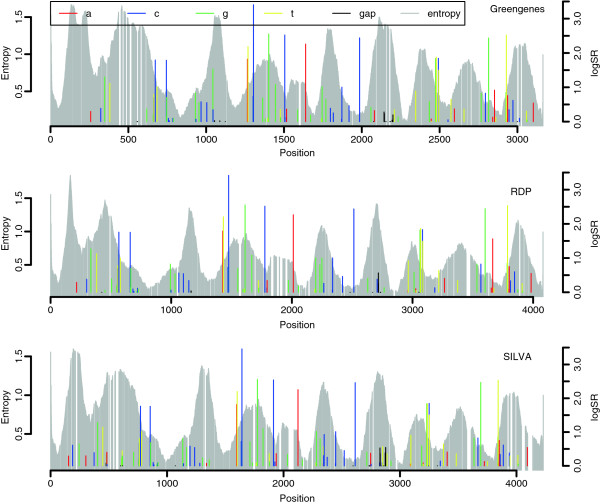
**Selected discriminative sites.** The selected sites for classification of phyla are plotted as coloured bars along the alignments. The height of a bar is the log-transformed SR-score (right hand vertical axis). The colors indicate which of the symbols A, C, G, T or - have the dominating discriminating power at the respective site, see legend. The grey bars in the background show the smoothed entropy values (left hand vertical axis) at each site as in Figure 2.

Figure 3 is an illustration of the selected discriminative sites together with the smoothed entropy from Figure 2. For all three alignments we ended up with 50 selected sites. The coloured bars indicate the selected sites. The height of a bar is the (log-transformed) SR-score, i.e. the tallest bars indicate the most discriminative sites. The color shows which symbol had the largest discriminatory power at the respective site. As an example, the leftmost bar is red, meaning the majority (but not necessarily all) of the information at this site is connected to whether a sequence has an A or not an A at this position. The three panels in Figure 3 are the results for the three different alignments. Despite the differences between the alignments, the selected sites are remarkably similar with respect to the variable and conserved regions. The largest single SR-score is the site indicated by the tallest blue bar. If we compare its location to the entropy in the background, we find it at the left hand side of region V4 in all three cases. Since both relative location and the colors of the selected sites are similar for the three panels, the results of the selection algorithm are stable with respect to the different alignments.

The first impression given by Figure 3 is that the selected sites are scattered across almost the entire alignment, there are no specific regions where they tend to cluster. As shown in Figure 2 we can identify the nine variable regions in each of the three alignments. By manual inspection we found their boundaries, and Table 2 shows the number of selected sites in each. Most notably is that around half of the 50 selected sites are outside the variable regions. The variable regions cover roughly half of all the positions in the alignments, hence a selected discriminative site is just as likely to occur outside as inside of these regions. From Table 2 we also see that regions V2 and V4 contain many selected sites, while V8 has none in all three cases. Regions differ in width, and V4 has most selected sites per position.

**Table 2:** Overview of the positions of the selected sites

**Database**	**V1**	**V2**	**V3**	**V4**	**V5**	**V6**	**V7**	**V8**	**V9**	**Outside**
Greengenes	0	7	2	6	3	1	3	0	2	26
RDP	0	7	1	6	3	3	1	0	1	28
SILVA	2	6	1	6	4	3	1	0	1	26

Each cell shows the number of selected sites for phylum classification found inside the variable regions V1-V9 for the three data sets. The rightmost column, named Outside, are the number of selected sites outside the variable regions. The total number of selected sites are 50 in each case.

**Figure 4:**
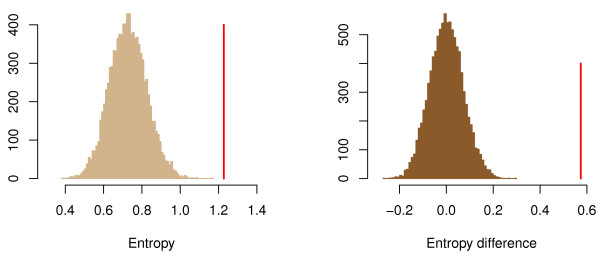
**Entropy of selected sites.** The left panel shows that mean entropy of the selected sites compared to random samples. The vertical red bar marks the mean entropy of the 50 selected sites, at 1.23. The histogram is constructed by sampling 50 random positions, computing their mean entropy, and repeating this 10 000 times. The right panel shows the mean difference between the entropy of a selected site and its 20 neighbors (10 on each side). Again the red bar marks this difference for the 50 selected sites and the histogram displays the same difference for 50 sites sampled at random, repeated 10 000 times. This figure is based on the Greengenes data, but the RDP and SILVA data gave similar results.

Even if selected sites are both inside and outside of variable regions, their actual site-specific entropy from eq. (1) are in all cases significantly above the average entropy for the entire alignment. This was tested by a simple permutation test, and the results are displayed in the left panel of Figure 4. The histogram shows the average entropy for 50 randomly sampled sites (repeated 10 000 times) in the Greengenes alignment, and the red bar marks the average for the 50 sites selected by PLS. Clearly, the selected sites have a mean entropy (1.23) which is much larger than what we expect at random (histogram), giving a p-value *p*<0.0001 here. The point is that selected sites have high entropy, but are not necessarily located in high-entropy regions. In fact, they tend to have much higher entropy than their surrounding sites, which is shown in the right panel of Figure 4. Here we computed the difference between the entropy of a selected site and its 10 neighbouring sites at each side. For the Greengenes data this resulted in the average difference 0.57 marked by the red bar. The histogram is again the result of a permutation test (10 000 permutations) where the same difference has been computed for randomly sampled sites. The results of Figure 4 were very similar for the RDP and Silva alignments, and are not shown here.

**Figure 5:**
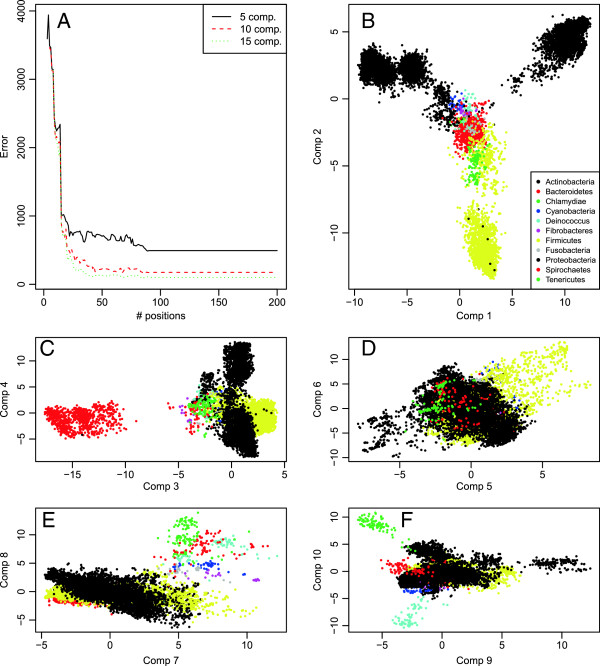
**Details of phylum classification.** Panel **A** shows how the number of mis-classifications drops as more and more sites are selected by the site selection algorithm. The error levels out at around 100 mis-classifications, and 50 selected sites seems to be enough to achieve this error rate. Panels **B-F** are PLS-plots of the sequence data, and the various panels show the same data from different perspectives. In panel **B** we plot the data in a coordinate system spanned by PLS-component 1 and 2, in panel **C** it is spanned by component 3 and 4 and so on. Each dot corresponds to a sequence, and the colors represent the true class label for each sequence, indicated by the legend in panel **B**. This figure is based on the Greengenes data, but the RDP and SILVA data gave similar results.

Figure 5 presents some detailed results for phylum classification based on the Greengenes alignment, again the results turned out similar for the RDP and SILVA alignment. Panel A (top left) shows how the number of mis-classifications decreases by including more selected variables, and converging at around 100 errors, giving an accuracy of over 99%. The other five panels visualize sequences in PLS-plots. Every point represents a sequence and the coordinate axes represent the optimal combinations selected by PLS (PLS components). Sequences located near each other are aligned similarly, at least in the discriminative sites. The colors represent the true classes (phyla). The first components separate the large classes, and it is not until the 10th component that smaller groups are separated. In panel B of Figure 4, we can see some obvious mis-classifications. Some black dots (supposedly Proteobacteria) are found in the center cloud of yellow (Firmicutes). This must be due to either alignment errors or sequences assigned to the wrong class from the beginning. In order to construct the huge alignments we use here, greedy algorithms of some kind are required. This means errors accumulate, and alignments of this size will most likely contain a substantial number of errors. Structure-based alignment methods should perform better for RNA-sequences. The RDP alignment we use here is based on the Infernal software [29], but still we find a number of mis-classifications. These errors constitutes a significant source of the classification errors we observe. In fact, the methods most frequently used for classification are those based on word-frequencies instead of alignments, e.g. the RDP-classifier [30], indicating that huge, monolithic alignments are quite poor data for classification purposes. However, when linking the classification to the location of conserved and variable regions, the use of alignments seems unavoidable.

From Figure 5 we see how the separation of the larger classes is more important than the smaller classes, since the first PLS-components are devoted to this. Each misclassification counts equally much, and separating larger classes will always reduce the total error more. This means the selected sites we find are those sites most important for separating the larger classes. The number of sequences in each class varies a lot in all available 16S data sets, e.g. see Figure 1. In this study we have only focused on the total error, and different results would be found if we focused only on the smaller classes.

**Figure 6:**
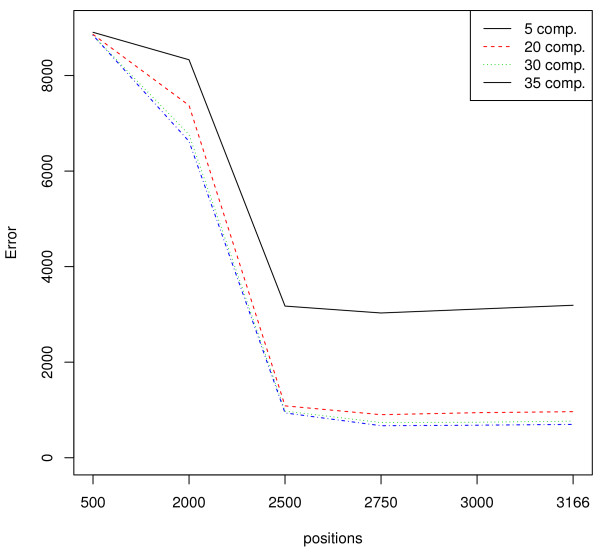
**Site selection for genus classification.** The figure illustrates how the number of mis-classifications drops by increased number of selected sites for genus classification. The error levels out at around 1000, and we need around 2500 sites to achieve this. This figure is based on the Greengenes data, but RDP and SILVA gave similar results.

Next, we repeated everything done so far, but using genus instead of phylum as class labels. This means we have 198 instead of 11 classes, making the separation much more difficult. In Figure 6 we show how the number of mis-classifications drops as we select more and more sites in the Greengenes alignment. We need to include many more sites than for phylum, and the classification error seems to level out after around 2500 selected sites, the remaining 600-700 sites do not provide further information about genus. Since around 80% of the sites are selected, it is obvious that the discriminating information in this case is not restricted to the variable regions. In fact, it tells us that in order to separate genera, we need to utilize almost every difference that can be found in the sequences regardless of where they are located. The error level we reach here, around 10% mis-classifications, is comparable to those reported by other studies on the genus level. This error rate and the number of selected sites indicates that a 16S based classification of genera means we are pushing the limit for how much information we can extract from the alignments of a single gene marker.

## Conclusion

The aim of this study was to investigate the dogma of 16S based classification, stating that the key information for separating classes is harboured in the variable regions of this marker. By using three different multiple alignments of the same sequence data, we implemented a supervised learning method to systematically search for discriminative sites without any constraints with respect to conservation. The selected sites came out remarkably similar for the three data sets, a sign of a stable selection despite the obvious differences between the three alignments.

Our first major finding is that the discriminative sites are not exclusively located in the variable regions. In fact, the nine variable regions are not even enriched with sites selected by our algorithm. Variable regions are important, but not more important than any other region. The second major finding is that discriminative sites are typically sites with high entropy located among neighbouring sites of much lower entropy. This seems like a logical outcome. Regions of lower entropy means some degree of conservation, and alignments tend to be more accurate in such regions. If a site inside such regions show a much larger variation, it is more likely this is due to real biology, not alignment errors.

We believe these findings should be taken into consideration when it comes to improving methods for 16S based classification of bacteria.

## Competing interests

The authors declare that they have no competing interests.

## Authors’ contributions

The project was initiated by LS and KHL. All authors have been involved in the development of the approach. KHL and HV did the programming. HV and LS drafted the manuscript. All authors have read and approved the final version.
